# Biological parameters, life table and thermal requirements of *Thaumastocoris peregrinus* (Heteroptera: Thaumastocoridae) at different temperatures

**DOI:** 10.1038/s41598-019-45663-5

**Published:** 2019-07-15

**Authors:** L. R. Barbosa, F. Santos, E. P. Soliman, A. P. Rodrigues, C. F. Wilcken, J. M. Campos, A. J. V. Zanuncio, J. C. Zanuncio

**Affiliations:** 1 Empresa Brasileira de Pesquisa Agropecuária- Embrapa Florestas, 83411-000 Colombo, Paraná Brazil; 2 Departamento de Entomologia e Acarologia, Escola Superior de Agricultura “Luiz de Queiroz”, Universidade de São Paulo, 13418-900 Piracicaba, São Paulo Brazil; 3 Departamento de Proteção Vegetal, Faculdade de Ciências Agronômicas, Universidade Estadual Paulista “Júlio de Mesquita Filho”, 18610-307 Botucatu, São Paulo Brazil; 4 Departamento de Fitotecnia, Universidade Federal de Viçosa, 36570-900 Viçosa, Minas Gerais Brazil; 5 Departamento de Engenharia Florestal, Universidade Federal de Viçosa, 36570-900 Viçosa, Minas Gerais Brazil; 6 Departamento de Entomologia/BIOAGRO, Universidade Federal de Viçosa, 36570-900 Viçosa, Minas Gerais Brazil

**Keywords:** Developmental biology, Invasive species

## Abstract

Temperature affects the development, population dynamics, reproduction and population size of insects. *Thaumastocoris peregrinus* Carpintero et Dellape (Heteroptera: Thaumastocoridae) is a eucalyptus pest. The objective of this study was to determine biological and life table parameters of *T*. *peregrinus* on *Eucalyptus benthamii* at five temperatures (18 °C; 22 °C; 25 °C; 27 °C and 30 °C) with a relative humidity (RH) of 70 ± 10% and photoperiod of 12 hours. The duration of each instar and the longevity of this insect were inversely proportional to the temperature, regardless of sex. The nymph stage of *T*. *peregrinus* was 36.4 days at 18 °C and 16.1 days at 30 °C. The pre-oviposition period was 5.1 days at 30 °C and 13.1 days at 18 °C and that of oviposition was 7.6 days at 30 °C and 51.2 days at 22 °C. The generation time (T) of *T*. *peregrinus* was 27.11 days at 22 °C and 8.22 days at 30 °C. Lower temperatures reduced the development and increased the life stage duration of *T*. *peregrinus*. Optimum temperatures for *T*. *peregrinus* development and reproduction were 18 and 25 °C, respectively.

## Introduction

The frequent introduction and establishment of exotic insect pests on eucalyptus plantations in Brazil are impacting and reducing productivity. The bronze bug *Thaumastocoris peregrinus* Carpintero & Dellapé (Hemiptera: Thaumastocoridae), an Australian eucalyptus pest was first recorded in Brazil in 2009^1^. At high infestations, this insect decreases the photosynthetic rate, leading to partial or total plant defoliation, and in some cases, plant death^2,3^.

Studies have focused on the biology^4–6^, chemical control^7^, chemical ecology^8,9^, morfology^10^, remote sensing for monitoring^11,12^ and biological control^13–16^ of this pest, aiming to minimize losses. However, the effect of temperature on the biological parameters of this species is not yet well known.

Ambient temperature is one of the most important abiotic factors affecting the survival, development rate, abundance, behavior and fitness of insects^17–20^. In fact, each insect species has an optimum temperature at which they thrive, with lower and upper limits for development^21,22^. High temperatures can decrease fecundity, hatching and survival of these organisms^23^, while low temperatures can affect the sex ratio (reduce male proportion), behavior, and population distribution of insects^24^.

The study of temperature in life-history variables, such as nymph development period, adult longevity and fecundity is crucial to the development of pest-management strategies^25^. The temperature decrease *Parapoynx crisonalis* (Lepidoptera: Pyralidae) life tables^26^ and *Brachmia macroscopa* (Lepidoptera: Gelechiidae) development and fecundity^27^. Thus, the objective of this study was to evaluate the effect of different temperatures on biological parameters of *T*. *peregrinus*.

## Results

### Nymph development

The nymph development period of *T*. *peregrinus* differed across temperatures (Kruskal-Wallis on ranks; df = 4, H = 168.42, P < 0.001) (Table 1). Furthermore, this parameter affected the duration of each instar (first-instar, Kruskal-Wallis on ranks; df = 4, H = 219.31, P < 0.001; second instar, Kruskal-Wallis on ranks; df = 4, H = 198.67, P < 0.001; third instar, Kruskal-Wallis on ranks; df = 4, H = 172.49, P < 0.001; fourth instar, Kruskal-Wallis on ranks; df = 4, H = 134.77, P < 0.001; and fifth instar, Kruskal-Wallis on ranks; df = 4, H = 126.4, P < 0.001) of this insect.

**Table 1:** Duration (mean ± SE) of each instar and of the nymph period (days) (Ny-Ad.) of *Thaumastocoris peregrinus* (Heteroptera: Thaumastocoridae) reared at different temperatures, RH of 60 ± 10% and photoperiod 12:12 (L: D) h.

°C	First instar	Second	Third	Fourth	Fifth	Ny-Ad.
18 °C	6.79 ± 0.23a	6.30 ± 0.21a	6.02 ± 0.23a	6.93 ± 0.22ª	10.42 ± 0.31a	36.37 ± 0.26ª
	n = 84	n = 75	n = 71	n = 63	n = 58	n = 52
22 °C	6.46 ± 0.14a	5.59 ± 0.17a	5.97 ± 0.21a	6.02 ± 0.26b	8.78 ± 0.29b	32.47 ± 0.33b
	n = 93	n = 82	n = 70	n = 63	n = 51	n = 36
25 °C	4.561 ± 0.13b	3.82 ± 0.11b	4.05 ± 0.11b	4.24 ± 0.09c	6.15 ± 0.14c	22.69 ± 0.16c
	n = 87	n = 81	n = 74	n = 65	n = 60	n = 52
27 °C	4.07 ± 0.12c	3.45 ± 0.11b	3.58 ± 0.16c	3.90 ± 0.14c	5.34 ± 0.24 cd	20.0 ± 0.25d
	n = 78	n = 71	n = 60	n = 52	n = 41	n = 33
30 °C	3.45 ± 0.11d	2.81 ± 0.09c	2.69 ± 0.12d	2.97 ± 0.23d	4.43 ± 0.26d	16.13 ± 0.20e
	n = 81	n = 77	n = 58	n = 45	n = 35	n = 23

Means followed by the same letter per line do not differ by the Tukey test (p ≤ 0.05).

### Adult reproduction and longevity

The pre-oviposition period of *T*. *peregrinus* decreased linearly with increased temperature, ranging from 13 (18 °C) to 5 (30 °C) days (Table 2). The fertility of this insect was similar at 22 °C (64 eggs), 18 °C (45.9 eggs), 25 °C (58.1) and 27 °C (49.1), while it was lower at 30 °C (22 eggs) (Table 2).

**Table 2:** Duration (mean ± SE) of the pre-oviposition (Preov.) and oviposition (Ovip.) (days), eggs per female (Eggs/female), eggs/female/day (Eggs/fem./day) and female (Fem. Long.) and male (Male Long.) longevity of *Thaumastocoris peregrinus* (Heteroptera: Thaumastocoridae) males and females at different temperatures, RH of 60 ± 10% and photoperiod 24:12 (L: D) h.

°C	18 °C	22 °C	25 °C	27 °C	30 °C
N	20	11	20	13	8
Preov	13.10 ± 0.61ª	9.09 ± 0.41^b^	6.20 ± 0.24^c^	6.31 ± 0.59^c^	5.13 ± 0.55^c^
Ovip. (days)	36.3 ± 3.8^ab^	51.2 ± 6.4^b^	29.9 ± 6.4^a^	21.5 ± 3.4^a^	7.6 ± 3.4^c^
Eggs/female	45.9 ± 4.6^ab^	64.0 ± 9.08^b^	58.1 ± 8.5^ab^	49.08 ± 9.18^ab^	22.8 ± 12.5^a^
Eggs/fem./day	1.1 ± 0.1ª	1.2 ± 0.09^ab^	1.6 ± 0.1^bc^	1.9 ± 0.19^c^	1.8 ± 0.4^ac^
Fem. Long. (days)	41.84 ± 3.9^cd^	53.6 ± 6.2^b^	34.3 ± 3.6^bc^	24.69 ± 3.11^ab^	10.4 ± 3.4^a^
Male Long. (days)	57.4 ± 3.4c	54.1 ± 7.0^c^	35.4 ± 1.8^b^	32.62 ± 3.29^b^	11.3 ± 2.9^a^
Sex ratio*	0.48ª	0.58ª	0.48ª	0.53^a^	0.61^a^

Means followed by the same letter per line do not differ by Tukey test (p ≤ 0.05).

Female longevity of *T*. *peregrinus* was longest at 22 °C (53 days) and that of males at 18 to 22 °C (57 and 54 days, respectively) (Table 2). Temperature did not affect the sex ratio of this insect (GLM-binomial: χ2190 = 1.96, p = 0.74) (Table 2).

### Survival analysis

**Figure 1:**
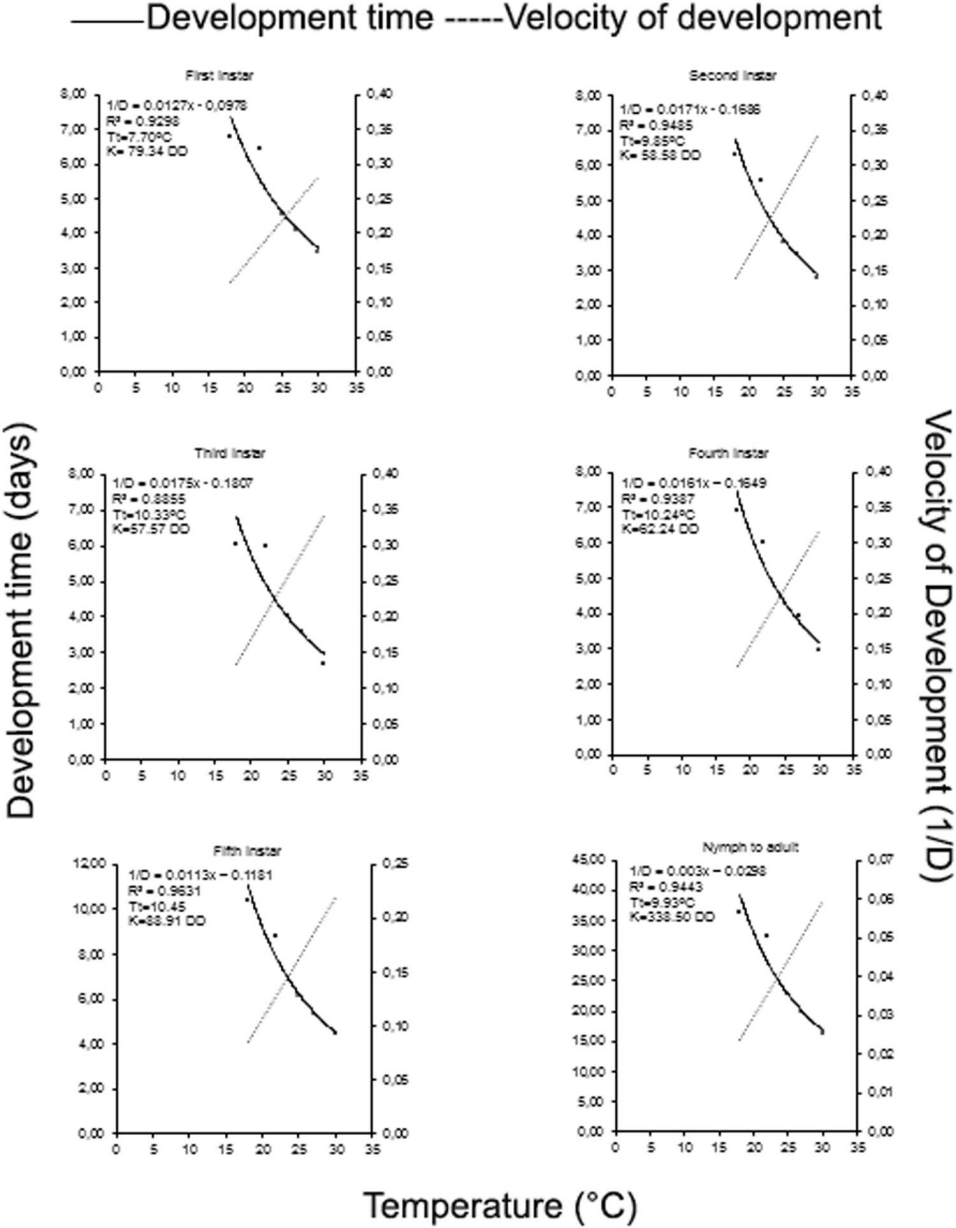
Relationship between temperature, development speed (1/days) of nymph and period of nymph-adult of *Thaumastocoris peregrinus* (Heteroptera: Thaumastocoridae), RH of 60 ± 10% and photoperiod 12:12 (L: D) h.____Development time (Days)  Velocity of development (1/D).

Temperature affected the survival rates of *T*. *peregrinus* nymphs (Mantel-Haenzel Test; χ^2^ = 53.6, P < 0·0001) (Fig. 1A), females (Mantel-Haenzel Test; χ^2^ = 60.9, P < 0·0001) (Fig. 1B), and males (Mantel-Haenzel Test; χ^2^ = 103, P < 0.0001) (Fig. 1C).

**Figure 2:**
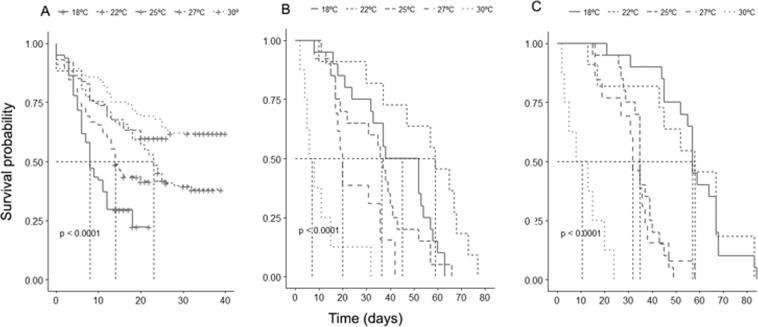
Kaplan–Meier survival curve for *Thaumastocoris peregrinus* (Heteroptera: Thaumastocoridae) nymph and adult at different temperatures. (**A**) Nymph stage; (**B**) Female adults; **C**) Male adults.

Survival analysis using the Cox’s Proportional Hazards model showed a higher death risk (hazard ratio; HR) for nymphs and adults (females and males) of *T*. *peregrinus* as temperature increased (Table 3) and (Fig. 2).

**Table 3:** Relative risk estimates for *Thaumastocoris peregrinus* (Heteroptera: Thaumastocoridae) reared at different temperatures using multivariable Cox regression analysis.

°C		HR	95% CI		z- value*
			Lower	Upper	
	18 °C	Reference			
	22 °C	1.85	1.20	2.87	**0.005**
	25 °C	1.39	0.85	2.27	0.1806
Nymph	27 °C	2.53	1.58	4.04	**<0.001**
	30 °C	4.16	2.64	6.56	**<0.001**
	18 °C	Reference			
	22 °C	0.33	0.13	0.81	**0.016**
Female	25 °C	1.41	0.75	2.65	0.283
	27 °C	3.05	1.43	6.50	**0.003**
	30 °C	12.61	4.96	32.07	**<0.001**
	18 °C	Reference			
	22 °C	1.03	0.49	2.18	0.923
Male	25 °C	6.87	3.11	15.19	**<0.001**
	27 °C	6.84	2.97	14.82	**<0.001**
	30 °C	100.3	29.24	344.18	**<0.001**

*Wald statistic value (z). Abbreviations: Hazard Ratio (HR); Confidence Interval (CI).

### Threshold development and thermal constants

The linear regression estimative for the temperature limit of *T*. *peregrinus* first, second, third, fourth and fifth instars was 7.70, 9.85, 10.33, 10.24 and 10.45 °C, respectively (Fig. 1). The *T*. *peregrinus* thermal constant (K) per instar was 79.34 degree-day (DD) (first), 58.58 DD (second), 55.57 DD (third), 62.24 DD (fourth) and 88.91 DD (fifth). The accumulated temperature for the nymph-to-adult period of this insect was 338.50 DD, with a temperature limit of 9.93 °C (Fig. 1).

### Life table

The net reproductive rate (R_0_) of *T*. *peregrinus* was higher at 25 °C (6.39) and 18 °C (4.45), the latter being similar to that at 22 °C (4.00). The net reproduction rate was lower at 30 °C (0.13). The generation time (T) of *T*. *peregrinus* varied between 27.11 days at 22 °C to 8.22 at 30 °C, and the intrinsic growth rate (rm) and finite increase (λ) of *T*. *peregrinus* were higher at 25 °C (0.046 and 1.047, respectively) and lower at 30 °C (0.084 and 0.919, respectively) (Table 4).

**Table 4:** Fertility life table of *Thaumastocoris peregrinus* (Heteroptera: Thaumastocoridae) reared at different temperatures (Temp.), RH of 60 ± 10% and photoperiod 24:12 (L: D) h.

Temp.	Life table parameters*			
	*Ro*	T (d)	r_m_	Λ
18 °C	4.56 ± 0.46ab	25.66 ± 1.413a	0.027 ± 0.001a	1.027 ± 0.002a
22 °C	4.00 ± 0.595 ab	27.11 ± 2.383 a	0.025 ± 0.002 a	1.026 ± 0.002 a
25 °C	6.39 ± 0.856 b	15.19 ± ± 0.602 bc	0.046 ± 0.002 b	1.047 ± 0.002 bc
27 °C	2.71 ± 0.510 cd	22.79 ± 3.668 ac	0.030 ± 0.005 a	1.031 ± ± 0.005 a
30 °C	0.13 ± 0.074 e	−8.22 ± 4.169 d	−0.084 ± 0.063 a	0.919 ± 0.006 ac

Means followed by the same letter per column do not differ by Tukey test (p ≤ 0.05). *Reproductive net rate (R_0_), mean generation time (T), innate ability to increase (rm), finite rate of increase (λ).

## Discussion

Temperature strongly influences insect development in both single generation progeny and in organisms that are established and successfully continued for multiple generations^28^. *Thaumastocoris peregrinus* development and reproduction reinforces the temperature effect on insects^29^, with the duration of its juvenile stage decreasing as temperature increases, as found for *Corythucha ciliate* (Say) (Hemiptera: Tingidae) and *Loxostege sticticalis* (L.) (Lepidoptera: Crambidae)^30^.

The shorter duration of each instar and of the adult period of *T*. *peregrinus* at higher temperatures is due to increased metabolism, food intake and energy, allowing the insect to reach the next stage^31,32^. Other factors, such as poor food quality^9,33,34^, decreased the survival and/or insect growth rate^35^. The ladybird *Harmonia axyridis* (Pallas) (Coleoptera: Coccinellidae)^36,37^, the dragonfly *Ischnura verticalis* (Odonata: Coenagrionidae)^38^, and the locust *Romalea microptera* (Orthoptera: Romaleidae)^39^ had shorter juvenile stages at increased temperatures.

*Thaumastocoris peregrinus* had a shorter pre-oviposition period with increased temperature, indicating the effect of this parameter on this organism. This is also reflected in the mating and egg laying of *T*. *peregrinus* as reported for *Phenacoccus madeirensis* Green (Hemiptera: Pseudococcidae)^40^ and *Leptocoris achinensis* (Dallas) (Hemiptera: Alydidae)^41^ and food/temperature and bioecology interactions^42^ as reported for *Cimex lectularius* (Linnaeus 1758; Hemiptera: Cimicidae)^43^. The number of eggs per *T*. *peregrinus* female at 26 °C on *Eucalyptus urophylla* x *Eucalyptus camaldulensis*^5^ and *Eucalyptus scoparia* at different temperatures^4^ and with *E*. *tereticornis* at 25 °C^44^ varied within certain limits^45^. The longer pre-oviposition period, at least for some *T*. *peregrinus* females at lower temperatures could be due to the longer time required for this predator to develop its ovary^46^.

*Thaumastocoris peregrinus* female and male longevity was increased at temperatures between 18 to 22 °C, which could be due to reduced metabolic processes at lower temperatures, affecting development and life history^47^. At low metabolic rates, certain physiological processes are suppressed, for example reproduction^48^, in order to maintain more crucial processes for survival. The effect of low temperatures on longevity have been reported for *Monosteira unicostata* Mulsant & Rey 1852 (Hemiptera: Tingidae) and *Cleruchoides noackae* Lin & Huber, 2007 (Hymenoptera: Mymaridae)^49,50^.

The optimal temperature range for *T*. *peregrinus* development and reproduction between 25 and 30 °C was similar to those reported for egg, nymph and egg-adult periods, respectively, for this bug^44,51^, as well as for *Nezara viridula* (L.) (Hemiptera: Pentatomidae) collected in soybean fields at climatically different locations^28^. The linear increase in the ratio between instars and of the adult stage duration of *T*. *peregrinus* (1/D) confirms the energy gain for its physiological processes^52^.

The low survival at high temperatures indicates a phenotypic plasticity for *T*. *peregrinus* in different environments^53^.

The higher thermal constant of *T*. *peregrinus* nymph development, 338.50 DD with a minimum of 9 °C shows the impact of low temperatures on this insect^51,54^. This result was also observed for *Axinoscymnus cardilobus* (Ren and Pang) (Coleoptera: Coccinellidae), with 204 DD; it took 67 days at a minimum of 9.07 °C to complete one generation, while this was 120 days^55^ at 17 °C. However, the accumulated temperature for the nymph-to-adult period of *T*. *peregrinus*, with 395.43 DD with a temperature limit of 9.93 °C shows its high adaptive potential. This species needed 905.65 DD in Canberra, Australia, to complete a generation and survived at temperatures below 1.5 °C, with adults recovering at higher temperatures^51^.

The e R_0_, rm, T and λ of *T*. *peregrinus* showed shorter development periods and higher growth rates with increased temperature, similar to that reported for *Megacopta cribraria* (F.) (Hemiptera: Plastaspidae)^56^ and *Jakowleffia setulosa* (Jakovley, 1874) (Hemiptera: Lygaeidae)^57^. These characteristics are important to understand the impact of temperature on insect growth, survival, reproduction and population increase^58,59^. This is necessary because the energy generated by the anabolism and catabolism metabolic processes for insect growth and reproduction depends on the environmental temperature^60^.

The environmental temperature affected the development, fertility, longevity and mortality of *T*. *peregrinus*. Thus, the definition of thermal requirements for *T*. *peregrinus* can assist traditional techniques in managing this pest. As well, this important data can be used in simulating population dynamics, monitoring, population peaks, occurrence, ecological zoning and modeling in order to manage this pest.

## Material and Methods

### Insect rearing and temperature conditions

The experiments were conducted at the Forest Entomology Laboratory of Embrapa Florestas in Colombo, Paraná state, Brazil. *Thaumastocoris peregrinus* was reared in the laboratory at 24 ± 2 °C, 60 ± 10% RH, and a photoperiod of 12:12 h L:D on bouquets of *Eucalyptus benthamii* Maiden & Cambage (Myrtales: Myrtaceae) branches. The branches were fixed in a piece of foam to prevent drowning the insects in a 500-mL glass flask filled with water^61,62^. The effect of temperature on various biological parameters of *T*. *peregrinus* was evaluated at five constant temperatures (18, 22, 25, 27 and 30 ± 2 °C) with a photoperiod of 12:12 L: D and RH 70 ± 10% in climatic chambers (BOD Specification: Type B.O.D M.S.Mistura; model MSM 011/G; SERIES 1002.0157, Volts 220, W700).

### Nymph development

Newly hatched *T*. *peregrinus* nymphs were individually placed in acrylic plates (2.8 cm diameter × 1.5 cm) with a *Eucalyptus benthamii* fresh leaf disk (2.1 cm diameter) with its petiole introduced in a hydrogel layer (hydroplan-EB/HyC, SNF SA Floger) to maintain the leaf turgor. The eucalyptus leaf discs were replaced every two days. The duration and viability of *T*. *peregrinus* instars were assessed daily. Instar changes were evaluated based on the exuvia presence. Survival was evaluated in relation to the number of live individuals beginning each instar.

### Adult reproduction and longevity

*Thaumastocoris peregrinus* adults (<24 h old) were sexed based on its morphological characteristics^6^. A couple of this insect was placed per Petri dish (5.0 cm in diameter) with a fresh *E*. *benthamii* leaf disc (4.9 cm diameter). The pre-oviposition (female emergence to the first egg laying) and oviposition period, fecundity (number of eggs per female per day), longevity and mortality of *T*. *peregrinus* were evaluated. The males were not replaced. Mortality data were used to calculate longevity. Females were maintained until death, and egg numbers were use in the analysis.

### Development thresholds and thermal constants

The temperature development threshold (Tt) and thermal constant (K) of *T*. *peregrinus* were estimated using the hyperbole method^63^, based on the duration of the different instars, the nymph stage and the egg-adult period at 18, 22, 25, 27 and 30 ± 2 °C. The *T*. *peregrinus* instar development rate and nymph-to-adult period was regressed against temperature using a linear equation given by the formula: *1/D* = *a* + *bT*, where, 1/D is the insect development time (*D*) in days, and *T* is the temperature (°C). The intercept ratio over the slope of the regression line corresponds to the threshold temperature (Tt) and the thermal constant (K) was estimated by taking the inverse of the slope (1/b)^64^.

### Life table analysis

The *T*. *peregrinus* fertility life table at each temperature was built with specific survival at age x (lx), specific fertility (m_x_) and number of offspring reaching the age x in the next generation (l_x_m_x_). These data were used to calculate the net reproductive rate (R_0_), time between generations (T), innate ability to increase (r_m_) and finite rate of increase (λ) of this insect^65^.

### Biological parameter analysis

All data were first analyzed using the Shapiro-Wilk and Bartlett tests to determine data normality and homogeneity. The data related to each instar duration and of the nymph-to-adult period did not conform to normality, even after log transformation. Therefore, the comparisons were made using the non-parametric Kruskal-Wallis test. Pre-oviposition, oviposition, fecundity, oviposition rate and female and male longevity data were normally distributed, and thus they were analyzed using a linear model followed by a post hoc pairwise comparisons performed using Tukey HSD test (function glht, package multcomp)^66^. Sex ratio was analyzed using a generalized linear model (GLM) assuming binomial distribution^67,68^. The analyses were performed with the software R, version 3.3.2. The fertility life table was analyzed by Jackknife and the averages compared by Student’s t-test using the software SAS version 9.1^69^.

### Survival analysis

Survival curves were fitted and analysed using Kaplan-Meier survival probabilities (R version 3.3.2, “*survival*”, “survminer” packages)^70,71^ followed by a pairwise comparisons Mantel-Haenszel Test (Log-Rank test) and Cox Proportional-Hazard Model (PH Model). The data evaluated at 30 °C was used as the reference for the other treatments (temperatures) on Multivariate Cox regression. Individuals who did not die by the end of the nymph period were censored (0 = death event did not occur; 1 = death event occurred). The adult individuals were not censored, because the experiment finished with the death of all insects (females and males).
