# Author Correction: Biological parameters, life table and thermal requirements of *Thaumastocoris peregrinus* (Heteroptera: Thaumastocoridae) at different temperatures

**DOI:** 10.1038/s41598-020-57955-2

**Published:** 2020-01-16

**Authors:** L. R. Barbosa, F. Santos, E. P. Soliman, A. P. Rodrigues, C. F. Wilcken, J. M. Campos, A. J. V. Zanuncio, J. C. Zanuncio

**Affiliations:** 1 Empresa Brasileira de Pesquisa Agropecuária- Embrapa Florestas, 83411-000 Colombo, Paraná Brazil; 2 Departamento de Entomologia e Acarologia, Escola Superior de Agricultura “Luiz de Queiroz”, Universidade de São Paulo, 13418-900 Piracicaba, São Paulo Brazil; 3 Departamento de Proteção Vegetal, Faculdade de Ciências Agronômicas, Universidade Estadual Paulista “Júlio de Mesquita Filho”, 18610-307 Botucatu, São Paulo Brazil; 4 Departamento de Fitotecnia, Universidade Federal de Viçosa, 36570-900 Viçosa, Minas Gerais Brazil; 5 Departamento de Engenharia Florestal, Universidade Federal de Viçosa, 36570-900 Viçosa, Minas Gerais Brazil; 6 Departamento de Entomologia/BIOAGRO, Universidade Federal de Viçosa, 36570-900 Viçosa, Minas Gerais Brazil

Correction to: *Scientific Reports* 10.1038/s41598-019-45663-5, published online 15 July 2019

This Article contains errors.

The authors had originally considered only the period of oviposition in the analyses. However, for the longevity analyses it is important that both pre-oviposition and oviposition data is used. The authors re-did these analyses using both sets of data. This affects the female longitudinal results in Table 2 and female risk estimates in in Table 3, as well as the survival curve for female adults displayed in Figure 2b.

The correct versions of Tables 2 and 3 appear below as Tables 1 and 2. The correct version of Figure 2b appears below as Figure 1.

**Table 1:** Duration (mean ± SE) of the pre-oviposition (Preov.) and oviposition (Ovip.) (days), eggs per female (Eggs/female), eggs/female/day (Eggs/day) and female (Fem. Long.) and male (Male Long.) longevity of *Thaumastocoris peregrinus* (Heteroptera: Thaumastocoridae) males and females at different temperatures, RH of 60 ± 10% and photoperiod 24:12 (L: D) h.

°C	18 °C	22 °C	25 °C	27 °C	30 °C
N	20	11	20	13	8
Preov	13.10 ± 0.61ª	9.09 ± 0.41b	6.20 ± 0.24c	6.31 ± 0.59c	5.13 ± 0.55c
Ovip. (days)	36.3 ± 3.8ab	51.2 ± 6.4b	29.9 ± 6.4a	21.5 ± 3.4a	7.6 ± 3.4c
Eggs/female	45.9 ± 4.6ab	64.0 ± 9.08b	58.1 ± 8.5ab	49.08 ± 9.18ab	22.8 ± 12.5a
Eggs/fem./day	1.1 ± 0.1a	1.2 ± 0.09ab	1.6 ± 0.1bc	1.9 ± 0.19c	1.8 ± 0.4ac
Fem.Long. (days)	54.50 ± 3.94a	62.73 ± 6.12a	40.50 ± 3.49b	31.00 ± 3.07bc	15.50 ± 3.36c
Male Long. (days)	57.4 ± 3.4c	54.1 ± 7.0c	35.4 ± 1.8b	32.62 ± 3.29b	11.3 ± 2.9a
Sex ratio*	0.48a	0.58a	0.48a	0.53a	0.61a

Means followed by the same letter per line do not differ by Tukey test (p ≤ 0.05).

**Table 2:** Relative risk estimates for *Thaumastocoris peregrinus* (Heteroptera: Thaumastocoridae) reared at different temperatures using multivariable Cox regression analysis.

	°C	HR	95% CI		z- value*
			Lower	Upper	
Nymph	18 °C	Reference			
	22 °C	1.85	1.20	2.87	**0.005**
	25 °C	1.39	0.85	2.27	0.1806
	27 °C	2.53	1.58	4.04	**<0.001**
	30 °C	4.16	2.64	6.56	**<0.001**
Female	18 °C	Reference			
	22 °C	0.47	0.20	1.07	0.072
	25 °C	2.50	1.29	4.85	0.006
	27 °C	4.92	2.21	10.94	**<0.001**
	30 °C	22.98	8.57	61.64	**<0.001**
Male	18 °C	Reference			
	22 °C	1.03	0.49	2.18	0.923
	25 °C	6.87	3.11	15.19	**<0.001**
	27 °C	6.84	2.97	14.82	**<0.001**
	30 °C	100.3	29.24	344.18	**<0.001**

*Wald statistic value (z). Abbreviations: Hazard Ratio (HR); Confidence Interval (CI).

**Figure 1:**
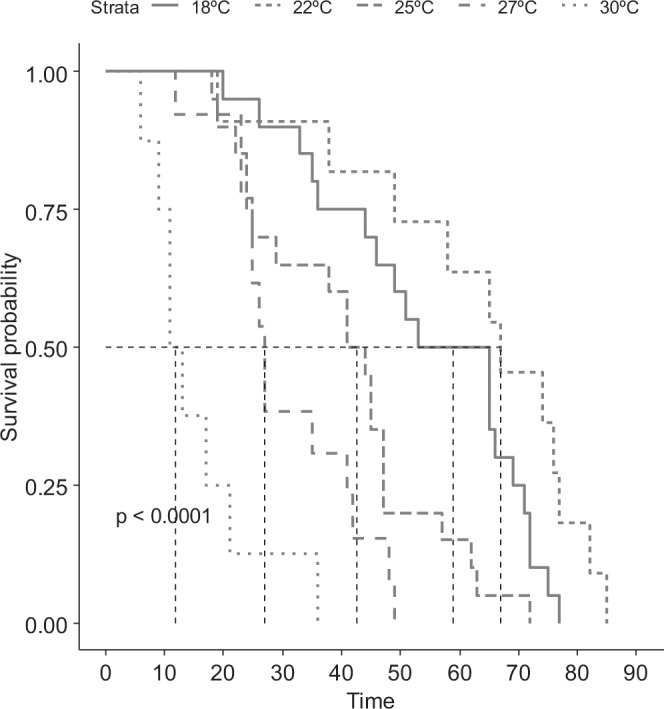
Kaplan–Meier survival curve for *Thaumastocoris peregrinus* (Heteroptera: Thaumastocoridae) female adults at different temperatures.

These changes do not affect conclusions of the Article.

